# COVID-19 in Japan during 2020-2022: Characteristics, responses, and implications for the health care system

**DOI:** 10.7189/jogh.12.03073

**Published:** 2022-10-14

**Authors:** Kenji Karako, Peipei Song, Yu Chen, Takashi Karako

**Affiliations:** 1 Department of Human and Engineered Environmental Studies, Graduate School of Frontier Sciences, The University of Tokyo, Chiba, Japan; 2 Center for Clinical Sciences, National Center for Global Health and Medicine, Tokyo, Japan; 3 International Health Care Center, National Center for Global Health and Medicine, Tokyo, Japan

The COVID-19 global pandemic of the past two years has changed the way humans behave, the way governments respond to crises, and placed a heavy burden on the health care systems of the countries in question. As 2022 begins, the world is experiencing a huge wave of infection with the Omicron variant of SARS-CoV-2 due to its higher transmissibility [1-3].

**Figure 1:**
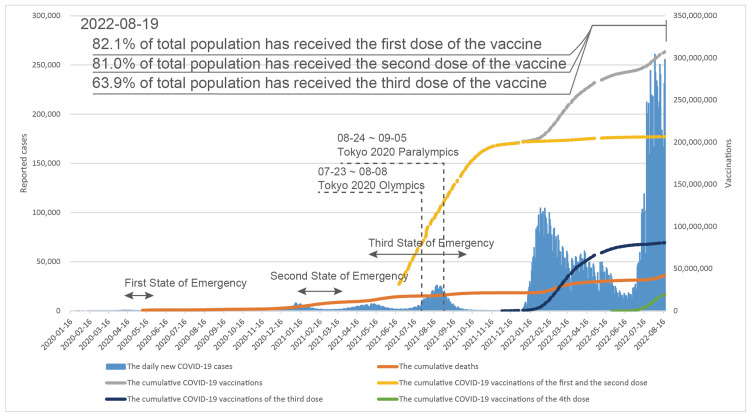
Number of reported COVID-19 cases, national response to the pandemic, and the vaccination campaign in Japan from 2020-2022. Data source: https://www.mhlw.go.jp/stf/covid-19/open-data.html, https://www.kantei.go.jp/jp/headline/kansensho/vaccine.html.

Globally, the number of new cases of COVID-19 reported on August 19, 2022 was 837 823 [4], of which 255 508 were reported in Japan [5], accounting for 30%. Worldwide, the progression of the COVID-19 epidemic and the response of the health care system in Japan have unique characteristics. This is particularly evident in declaring states of emergency during peaks with non-compulsory self-restraint requirements, the low mortality rate, the sharp decline in the number of cases after the impact of the Tokyo 2020 Olympic and Paralympic Games on the health care system, and the unprecedented rise in the number of infections due to the current spread of Omicron variant (**Figure 1**).

**Figure:**
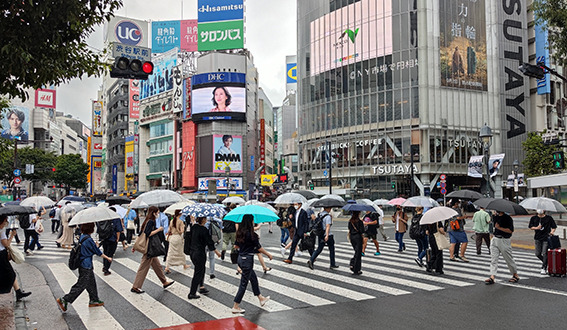
Photo: Shibuya Scramble Crossing, Tokyo (from the fourth author’s own collection, used with permission).

Since the first domestic case of COVID-19 transmission was reported on January 16, 2020, Japan has experienced six waves of the COVID-19 pandemic and is currently experiencing the seventh wave. Three states of emergency have been declared since April 2020, two of which were declared in 2021. During this period, Japan hosted the Tokyo 2020 Olympic (July 23-August 8, 2021) and Paralympic Games (August 24-September 5, 2021) and began a massive vaccination campaign. A record 5773 new cases were reported in Tokyo on August 13, and 25 975 new infections were reported nationwide on August 20, 2021. However, Japan appeared to have effectively controlled the spread of the disease by September 30, 2021, and from October 16, 2021 to January 2, 2022, the number of infected per day decreased to less than 500. After that, however, the number of infected increased rapidly due to the spread of Omicron variant. On July 20, 2022, the number of infected per day exceeded 200 000 for the first time, and the peak to date was reached on August 2, 2022 with 264 644 new infections [6]. As of August 19, 2022, Japan had reported a cumulative total of 16 423 053 infected and 36 234 deaths [5].

In Japan, COVID-19 is classified as a legally designated infectious disease (under the Infectious Diseases Act) from February 1, 2020 and the medical expenses of hospitalization has basically been covered by public funds [7-9]. The medical cost of treating COVID-19 is staggering, and this was especially true at the initial stages of the outbreak when clinical experience was lacking [10,11]. Contrary to expectations, however, annual medical expenses in Japan in 2020 decreased from the previous year. Annual medical expenses in Japan tended to increase each year until 2019. In 2020, however, when the outbreak of COVID-19 was confirmed, annual medical expenses decreased by 2.76% compared to the previous year, totalling JPY42.288 trillion (about US$ 402.743 billion, exchange rate for the same period) [12].

**Figure 2:**
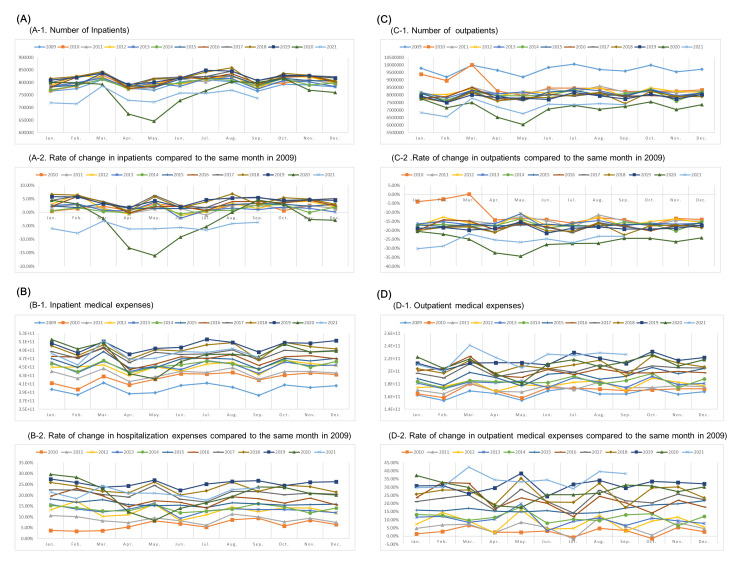
Changes in (**A**) number of inpatients, (**B**) medical expenses associated with hospitalization, (**C**) number of outpatients, and (**D**) medical expenses for outpatients in public hospitals in Japan from January 2009 to December 2021.

In order to ascertain the cause of the decrease in annual medical expenses during the epidemic and to examine COVID-19’s implications for the health care system, we analysed medical expenses from Japan’s universal health insurance system from 2009 to 2021. For the hospitalization and outpatient care at university hospitals, public hospitals, private hospitals, and clinics, the changes in the number of patients, the medical expenses, average medical expenses, and the average duration were analysed. The data analysis showed that the inpatient medical expenses at each type of medical facility in 2021 – which have decreased since the first state of emergency – have returned to their 2019 level. However, the number of inpatients in university hospitals and private hospitals in 2021 returned to the number in 2014, but the number of inpatients in public hospitals decreased by about 5% compared to 2009 (**Figure 2**). Public hospitals are particularly committed to providing COVID-19 patients with hospital beds, and the number of inpatients appears to have decreased accordingly. In addition, the number of outpatients in public hospitals in 2021 has not returned to the 2019 level.

Japan's basic policy for COVID-19 is to curb the outbreak of infection, maintain the medical system, and focus on dealing with the severely ill [13]. Based on this policy, Japan declared a state of emergency during peaks with non-compulsory self-restraint requirements, allocated hospital beds and at-home care depending on the symptoms of the infected, and preferentially supplied vaccines to the elderly. As of August 19, 2022, 82.1% of the total population nationwide has received the first dose of the vaccine, 81.0% has received the second dose, and 63.9% has received the third dose (**Figure 1**).

There has been an unprecedented increase in the number of infections due to the current spread of Omicron variant. As of August 19, 2022, the number of patients requiring inpatient care was 1 843 430 and the number discharged or released from treatment was 14 443 614 [5]. The current situation raises fears that the medical care provision system may soon become overwhelmed, including rising hospital bed occupancy rates for COVID-19 patients, increased numbers of emergency hospital admission difficulty cases, and shortages of health care workers. In addition, for patients with other diseases, hesitation to visit hospitals is occurring in substantial numbers due to the fear of COVID-19 [14], which will lead to worsening of the disease as symptoms are ignored.

The impact of maintaining a system to care for COVID-19 patients is evident in the data on medical expenses. Annual medical expenses in Japan in 2020 decreased for the first time during the past decade, mainly due to the decrease in the number of inpatients and outpatients during the COVID-19 outbreak since March 2020, especially in public hospitals. While priority must be given to treating severe COVID-19 patients in the current pandemic, there is an urgent need for the current Japanese health care system to adapt its response to ensure that routine treatment of other diseases proceeds smoothly and to enhance the response to moderate and mild COVID-19 cases.
